# Electrical Properties Tomography Based on *B*_1_ Maps in MRI: Principles, Applications and Challenges

**DOI:** 10.1109/TBME.2017.2725140

**Published:** 2017-08-21

**Authors:** Jiaen Liu, Yicun Wang, Ulrich Katscher, Bin He

**Affiliations:** Advanced MRI Section, Laboratory of Functional and Molecular Imaging, National Institute of Neurological Disorders and Stroke, National Institutes of Health, Bethesda, Maryland, 20892, USA; Department of Biomedical Engineering, University of Minnesota, Minnesota, 55455, USA; Philips Research Europe, Hamburg, 22335, Germany; Department of Biomedical Engineering and Institute for Engineering in Medicine, University of Minnesota, Minnesota, 55455, USA

**Keywords:** electrical properties, *B*_1_-mapping, electrical properties tomography, EPT, magnetic resonance imaging, MRI, Quantitative magnetic resonance imaging

## Abstract

Electrical properties (EPs), including conductivity and permittivity, are closely related to many fundamental properties of tissue and its pathological conditions. Noninvasively measuring the *in vivo* EPs of tissue can have an important impact on biomedical research and clinical applications. Among approaches developed for EP quantification and imaging, electrical properties tomography (EPT) is a promising technology that reconstructs EPs based on the *B*_1_ field distribution that can be measured in the radiofrequency range using an MRI device. In this article, we review the basic principle of EPT, reconstruction methods, biomedical applications including tumor imaging, and existing challenges. As an important application of EPT, the estimation of specific absorption rate (SAR) and its current development are discussed.

## I. Introduction

Electrical properties (EPs), also known as dielectric properties, including electrical conductivity (*σ*) and permittivity (*ε*), are fundamental properties of materials. Conductivity characterizes the capability to transfer electrical current inside the medium. Permittivity is directly related to the effect of electric polarization that happens when the electric charges of molecules separate to counteract the external electric field. In biological tissues, EPs are determined by the fundamental biophysical properties of the tissue, such as water content, ion concentration, molecular composition, fraction of intracellular space, permeability of the cell membrane, cellular structure, etc.[1]–[5] A unique frequency-dependent characteristic of biological EPs has been observed, with three major stages, also known as dispersions, at various frequencies [6]. This frequency dependency was explained in part by the nonconductive nature of cell membrane at low frequency and by the phenomenon of dielectric relaxation that happens on electrically polarized molecules – such as water and protein – and structures such as cell membrane in a time-varying electromagnetic field [4]. The frequency spectrum of biological EPs can be described by relaxation models with multiple relaxation time constants to incorporate various dispersions. These models include variants of Debye [3] or Cole-Cole [7] equations.

Electrical properties can potentially be used as biomarkers indicating the healthiness condition of tissue in clinical applications. Previous non-MRI-based (MRI: magnetic resonance imaging) studies have shown that various diseases cause local changes of EPs relative to the healthy nearby tissue. Among various cancers, breast cancerous tissue has been shown with the most different EPs compared to normal breast tissue [8], [9]. Benign breast tissue shows significantly different conductivity compared to malignant breast carcinoma [10]. Studies showed that cerebral ischemia could decrease conductivity up to 60% [11], [12], suggesting an effective method for differentiating ischemic and hemorrhagic stroke. Local ischemia and cell swelling that happen due to a focal seizure during epilepsy can also change the electrical properties [13].

Tissue EPs play an important role in biomedical research. They are useful for understanding the distribution of electromagnetic field inside tissue. For example, accurate EP models are needed to localize the internal electrical activities based on noninvasive electrophysiological recording over the body surface such as electroencephalography (EEG) [14]–[19] and electrocardiography (ECG) [20]–[24]. On the other hand, EPs are critical to estimate the distribution of electric current and electromagnetic power inside the body in applications utilizing electromagnetic stimulation for treatment, such as deep brain stimulation (DBS) to mitigate Parkinson’s disease symptom [25]–[27], transcranial direct current stimulation [28], transcranial magnetic stimulation (TMS) in neuropsychiatry [29], radiofrequency (RF) ablation to remove arrhythmic genesis foci [30] and RF hyperthermia in cancer treatment [31]. EP model of the body is also critical to quantify tissue heating induced by electromagnetic wave used in cell phones or MRI.

In the past decades, much effort has been made to map distribution of tissue EPs *in vivo*. Among related techniques, electrical impedance tomography (EIT) [32]–[36] has been well explored and developed to provide high temporal resolution. However, EIT requires mounting electrodes and injecting current into the sample but provides limited spatial resolution due to the ill-posed nature of the inverse problem. Magnetic resonance electrical impedance tomography (MREIT) [37]–[45] utilizes MRI to detect the magnetic field induced by the probing current, providing high spatial resolution, but requires safety-concerning current density to reach a sufficient signal-to-noise ratio (SNR) within an MRI scanner. Instead of injecting electrical current from surface electrodes which may suffer from the shielding effect of non-conductive tissue, magneto-acoustic tomography with magnetic induction (MAT-MI) was proposed [46], which uses magnetic induction and ultrasound measurement to obtain high spatial resolution [46]–[49]. However, what remains to be improved is the limited contrast in MAT-MI images due to electromagnetic interference.

**Fig. 1:**
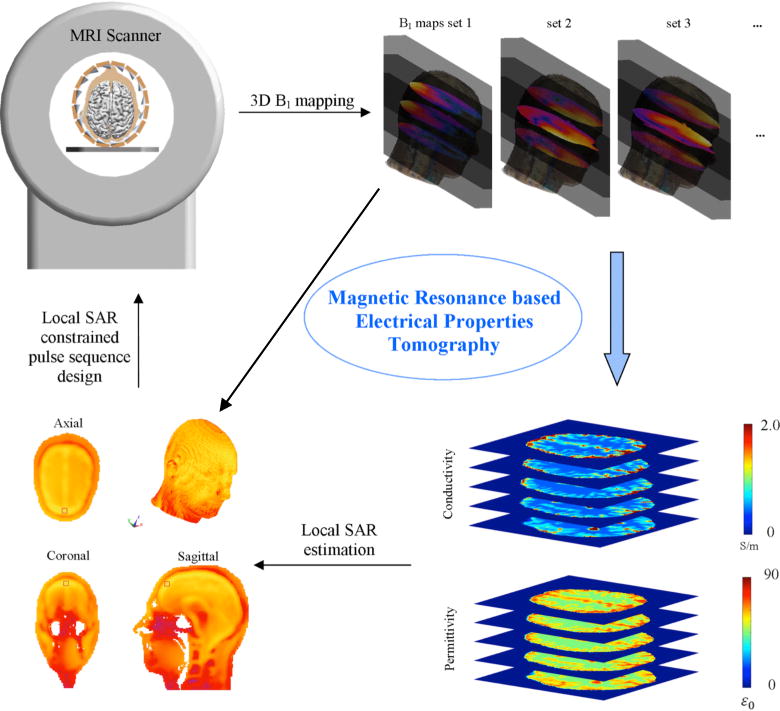
Schematic diagram illustrating electrical properties tomography and its application in imaging tissue electrical properties and predicting subject-specific absorption rate.

Recently, electrical properties tomography (EPT) was introduced as a non-invasive *in vivo* imaging approach to simultaneously map conductivity and permittivity at the resonant frequency of water proton using MRI [50]–[53]. As shown in Fig. 1, three-dimensional distribution of EPs can be reconstructed from measured maps of *B*_1_ field (also referred to as RF field) inside the sample. During an MRI scan, the *B*_1_ field is generated on an RF transmitting device (commonly known as an RF coil) for generating signals carrying information about the water protons in the body. The *B*_1_ field can be quantitatively mapped throughout the sample utilizing *B*_1_-mapping techniques. EPT reconstruction algorithms are developed based on the Maxwell’s equations, which address the distribution of electromagnetic field as a function of EP distribution in the sample. In principle, EPs can be reconstructed either voxel by voxel considering the distribution of *B*_1_ field within a voxel’s neighboring region or globally inside an entire volume utilizing global *B*_1_ information. EPT can achieve high spatial resolution and deep penetration depth without surface electrodes or current injection. An EPT scan is conducted similarly to existing MRI techniques, making it potentially applicable for clinical applications.

Sections II and III introduce the principle of EPT and review its reconstruction algorithms and corresponding experiment setups, respectively. Section IV provides a survey of the applications of EPT in which EPs were used as an imaging contrast to characterize diseased tissues.

Tissue heating due to power absorption from the applied RF field during an MRI scan is a safety concern and limiting factor for the development of high field MRI. Based on current standard (International Standard IEC 60601-2-33 2010), tissue heating is measured by the quantity of specific absorption rate (SAR), which must be estimated before scanning. Today’s MR scanners usually apply generic human models for local SAR determination, requiring large safety margins to account for potential deviations between the model and individual subjects. Optimally, local SAR would be estimated by a realistic, high resolution model based on *in situ* measurements of the individual subject, but the intimidating efforts required to set up such models currently prohibits their use in clinical routine. Moreover, an accurate SAR estimation requires modelling the involved transmit coil not in its ideal status but its operating status. Particularly for transmit arrays, which are promising for addressing *B*_1_ intensity variation at high fields (3 T or above) [54], [55], actual and ideal coil status might differ by loading effects, decoupling issues, dysfunction of single transmit channels, etc.

As an alternative to model-based SAR estimation, it was suggested to estimate SAR in the framework of EPT, i.e., by post-processing (complex) *B*_1_ maps as shown in Fig. 1. EPT-based SAR determination automatically includes the individual subject and the operating status of the transmit channel(s), overcoming corresponding problems of model-based SAR estimation and useful for designing the optimal *B*_1_ field for the individual subject. Thus, aside from diagnostic applications, EPT can play an important role in the framework of RF safety management. Section V outlines the background of EPT-based SAR determination and gives an overview of corresponding studies reported.

## II. Basic Principles of MR-based EPT

In this section, the concept of EPT will be introduced, the instrumental mechanism of measuring the *B*_1_ field using MRI for EPT reconstruction will be briefly reviewed, and the core equations deducing various reconstruction algorithms will be presented. Studies on anisotropic EPs are beyond the scope of this review.

### A. Relationship Between EPs and B_1_ Field

MRI employs a strong static magnetic field (*B*_0_ field) and the time-varying *B*_1_ field at the Larmor frequency to induce precession of the nuclear spins — of water proton within the scope of this review — about the direction of *B*_0_. In the view of classical mechanics, the assemble of spins can be represented by a vector of magnetization that is originally aligned with *B*_0_ and precesses about *B*_0_ after the RF excitation. The RF signal generated by the magnetization precession can be detected by receive RF coils.

The relationship between the distribution of the *B*_1_ field and the underlying EPs of the sample is governed by Maxwell’s equations [56], [57]. Faraday’s Law and Ampère’s Law in Maxwell’s equations describe the interactive relationship between the time-varying magnetic and electric field in a medium with a specific distribution of EPs and magnetic permeability (*μ*). Because the frequency spectrum of the applied *B*_1_ field is usually closely centered at the precessing frequency of the nuclear spins, the field can reasonably be treated to be time harmonic, i.e. oscillating at a single frequency. The time harmonic forms of Faraday’s Law and Ampère’s Law are

(1) ∇×E(r)=−jωB(r)

and

(2) $$\nabla \times B(r)=j\omega \mu \varepsilon_{c}E(r),$$

respectively. Here, **B**(**r**) and **E**(**r**) denote the spatially dependent component of the magnetic and electric field, respectively, in the Cartesian coordinate, $\varepsilon_{c}:=\varepsilon -i\frac{\sigma }{\omega }$ is known as the complex permittivity, **r** represents location and *ω* the oscillating angular frequency of the fields. In the rest of the paper, **r** will be eliminated since the derivatives only take place in space in the equations. Taking the curl operation on both sides of (2) and combining the result with (1), the relationship between **B** and the electromagnetic properties of the medium is

(3) $$-\nabla^{2}B=\omega^{2}\varepsilon_{c}\mu_{0}B+\nabla \varepsilon_{c}\times \frac{\nabla \times B}{\varepsilon_{c}}$$

where *μ* of tissue has been assumed to be equal to *μ*_0_ of the free space.

To be able to estimate EPs from MR images, the signals generating the images should carry some form of information about the *B*_1_ field. In general, the signals from a voxel in the sample induced by the transmit RF pulse and picked by a receive RF coil can be expressed as [58]

(4) $$S=\rho C\text{sin}(\alpha )e^{i\text{arg}(B_{1}^{+})}B_{1}^{-*}$$

where $B_{1}^{+}$ is traditionally known as the transmit *B*_1_ field inducing the precession of the magnetization, $B_{1}^{-}$ is the sensitivity of the RF coil to pick up the MR signals, also known as the receive *B*_1_ field, arg() denotes the phase of a complex number, ‘*’ indicates complex conjugate, *α* the angle between *B*_0_ and the magnetization after RF excitation (also known as the flip angle), *ρ* is the density of water protons and *C* represents a contrast term due to a combination of factors underlying an MR image, such as T1 and T2 relaxation, flip angle, etc. To accurately map *α*, *C* can either be designed to be insensitive to *α* [59] or manipulated to be sensitive to *α* in a specific fashion [60], [61].

Equation (4) shows that the MR signal is proportional to the sinusoid of flip angle and magnitude of $B_{1}^{-}$, and the signal is complex with its phase including the transceive phase, which is the sum of the phases of $B_{1}^{+}$ and $B_{1}^{-*}$. As a matter of fact, $B_{1}^{+}$ and $B_{1}^{-}$ are the components of the *B*_1_ field in the axial plane viewed in the positive and negative rotating frames, respectively [58]. The transformation from $B_{1}^{+}$ and $B_{1}^{-}$ in the rotating frames to *B*_x_ and *B*_y_ in the Cartesian frame can be formulated as [58]

(5) $$\left\{ \begin{array}{c} B_{1}^{+}=(B_{x}+iB_{y})/2\\ B_{1}^{-}={\left(B_{x}-iB_{y}\right)}^{*}/2 \end{array}\right.$$

which together with Gauss’s Law ∇ · **B** = 0 enables establishing the relationship between EPs and the MRI-visible $B_{1}^{+}$ and $B_{1}^{-}$as [62], [63]

(6) $$-\nabla^{2}B_{1}^{+}=\omega^{2}\varepsilon_{c}\mu_{0}B_{1}^{+}-\left(\frac{\partial B_{1}^{+}}{\partial x}-i\frac{\partial B_{1}^{+}}{\partial y}+\frac{1}{2}\frac{\partial B_{z}}{\partial z}\right)\left(g_{x}+ig_{y}\right)-\left(\frac{\partial B_{1}^{+}}{\partial z}-\frac{1}{2}\frac{\partial B_{z}}{\partial x}-i\frac{1}{2}\frac{\partial B_{z}}{\partial y}\right)g_{z}$$

and

(7) $$-\nabla^{2}B_{1}^{-*}=\omega^{2}\varepsilon_{c}\mu_{0}B_{1}^{-*}-\left(\frac{\partial B_{1}^{-*}}{\partial x}+i\frac{\partial B_{1}^{-*}}{\partial y}+\frac{1}{2}\frac{\partial B_{z}}{\partial z}\right)\left(g_{x}-ig_{y}\right)-\left(\frac{\partial B_{1}^{-*}}{\partial z}-\frac{1}{2}\frac{\partial B_{z}}{\partial x}+i\frac{1}{2}\frac{\partial B_{z}}{\partial y}\right)g_{z}$$

where **g**:= (*g_x_*,*g_y_*_,_ *g_z_*):= ∇ ln *ε_c_*. Equations (6) and (7) serve as the cornerstone to estimate EPs from measured *B*_1_ information. It should be noted that the *B_z_* component lies in the same direction as the *B*_0_ field and on the scale of 10^−6^ the latter. So *B_z_* does not contribute to MR signals significantly enough that it can be detected based on current MRI methods. For RF coils, of which the conductors are oriented in parallel with the *B*_0_ direction, *B_z_* can reasonably be assumed to be zero, and then, (6) and (7) become

(8) $$-\nabla^{2}B_{1}^{+}=\omega^{2}\varepsilon_{c}\mu_{0}B_{1}^{+}-\left(\frac{\partial B_{1}^{+}}{\partial x}-i\frac{\partial B_{1}^{+}}{\partial y}\right)\left(g_{x}+ig_{y}\right)-\frac{\partial B_{1}^{+}}{\partial z}g_{z}$$

and

(9) $$-\nabla^{2}B_{1}^{-*}=\omega^{2}\varepsilon_{c}\mu_{0}B_{1}^{-*}-\left(\frac{\partial B_{1}^{-*}}{\partial x}+i\frac{\partial B_{1}^{-*}}{\partial y}\right)\left(g_{x}-ig_{y}\right)-\frac{\partial B_{1}^{-*}}{\partial z}g_{z}$$.

Assuming the distribution of EPs is smooth so that the gradient term **g** can be ignored, (6) and (7) can be simplified into the Helmholtz equations as

(10) $$-\nabla^{2}B_{1}^{+}=\omega^{2}\varepsilon_{c}\mu_{0}B_{1}^{+}$$

and

(11) $$-\nabla^{2}B_{1}^{-*}=\omega^{2}\varepsilon_{c}\mu_{0}B_{1}^{-*}$$

where it shows that in homogeneous regions, EPs are solely determined by the distribution of the *B*_1_ components in the xy-plane which are in the MR-signal equation (4).

### B. B_1_-mapping

Equations (8) and (9) suggest that once the complex *B*_1_ field is mapped, EP distribution can be reconstructed. Conventionally, the so-called *B*_1_-mapping technique refers only to the measurement of the distribution of $\left|B_{1}^{+}\right|$ based on acquired MR images, but in the context of EPT reconstruction, both the magnitude and phase of $B_{1}^{+}$ or $B_{1}^{-}$ are relevant. A comprehensive review of different *B*_1_-mapping techniques is beyond the scope of this paper. In the following, only a few examples will be discussed to provide some basic ideas.

Since $\left|B_{1}^{+}\right|$ is proportional to the induced flip angle *α*, one strategy for mapping $\left|B_{1}^{+}\right|$ is to measure the distribution of *α*. Note that this linear relationship is normally valid because the RF pulse duration *τ* is much shorter than the T2* and T1 relaxation time of most tissues. For example, *α* can be calculated from the division of signals of two scans to eliminate other confounding factors, with *α* of one scan to be twice as much as that of the other and a long TR to minimize the dependency of *C* in (4) on *α* [59]. Another example of *α*-based *B*_1_ mapping is the so called actual flip angle imaging (AFI) [60], which exploits the signals from two alternating steady states established with two alternating TRs; the ratio of the signals or *C*s in the two states is a function of *α* and the ratio of TRs. In a more recent paper, a technique dubbed “dual refocusing echo acquisition mode” (DREAM) was proposed to calculate *α* based on the signals of stimulated echoes and free induction decay [61].

With the Bloch-Siegert shift-based approach, the phase of MR signals is utilized to derive $\left|B_{1}^{+}\right|$ when an off-resonance RF pulse, whose central frequency deviates from the Larmor frequency of the *B*_0_ field, induces a phase accumulation proportional to the square of $\left|B_{1}^{+}\right|$ [64], [65]. When the off-resonance frequency is significantly large, this relationship approximates to be linear. Two scans with symmetric off-resonance frequency shifts can be used to acquire the *B*_1_-related phase signal for estimating $\left|B_{1}^{+}\right|$.

## III. Reconstruction Methods

**Fig. 2:**
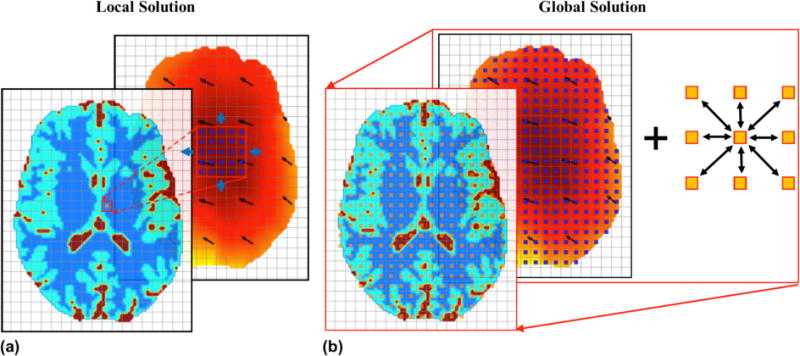
Illustration of local EPT reconstruction solutions in comparison with global EPT solutions. Using a local solution (a), EPs on each voxel are determined solely by the measured *B*_1_ data within its small neighborhood. On the other hand, a global solution (b) is obtained using all the voxels containing measurement information and by exploiting the inter-voxel relationship of EPs (indicated by arrows) to constrain the solution.

Methods for solving the EPT problem can generally be divided into local and global approaches. As shown in Fig. 2a, with the local solution, the measured *B*_1_ data in the voxels about a specific location is used to calculate the EPs at that location. The relationship of EPs between neighboring voxels is not utilized. On the other hand, the global solution is derived from measured *B*_1_ data within the entire region of interest (ROI) as shown in Fig. 2b, and the inter-voxel relationship of EPs is utilized either based on Maxwell’s equations or by introducing certain regularization strategies to improve the image quality.

In addition, among global solutions, methods can be categorized based on the direction of the calculation into forward and backward solutions. Employing a forward solution, the *B*_1_ field is updated iteratively by reducing the difference between the measured *B*_1_ data and the derived *B*_1_ distribution which is based on the calculated EPs, whereas a backward solution only involves calculation of EPs from the measured *B*_1_ data.

### A. Local Solutions

#### A.1. Helmholtz-based Methods Using a Birdcage RF Coil

**Fig. 3:**
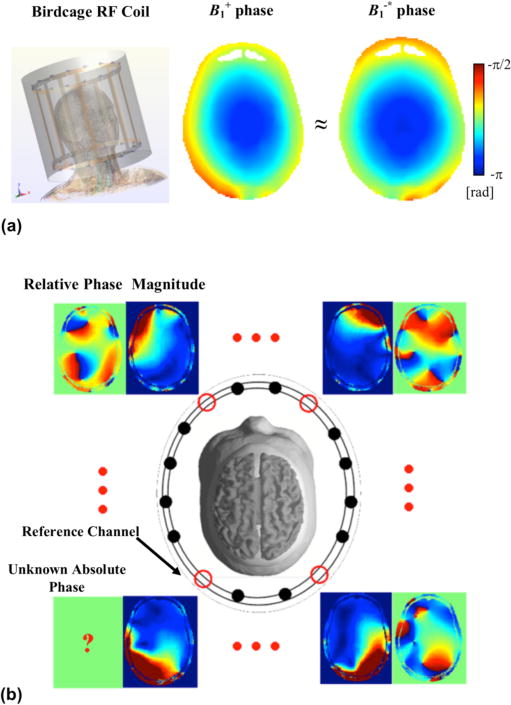
(a) Left: simulation model of a quadrature birdcage RF coil loaded with a realistic human head model at 3T; right: the simulated phase distributions of the transmit and receive *B*_1_ fields are approximately equal. (b) Diagram showing measurable *B*_1_ data, including magnitude and relative phase using a multi-channel transmit or receive RF coil array; the data shown is measured |*B*_1_^+^| and corresponding relative phase in a human subject at 7 T.

In clinical MRI setups at 1.5 or 3 T, the quadrature birdcage coil is the most commonly used transmit device due to its uniform $B_{1}^{+}$ coverage [66]. When the birdcage coil is used for transmitting and receiving, the absolute $B_{1}^{+}$ phase of the RF transmitter and the $B_{1}^{-*}$ phase of the receiver are approximately equal (“transceive-phase assumption”, TPA) as shown in Fig. 3a [51], [52], [67], [68]. Based on this empirical observation, the absolute $B_{1}^{+}$ phase can be estimated as half of the transceive phase after other phase contributions from off-resonance effect are removed. In Fig. 3b, the idea of quantitative estimation of absolute *B*_1_ phase using *B*_1_ information from multiple RF channels is illustrated and will be discussed in detail in Section A.2.

As can be seen in the Helmholtz equations (10) or (11), assuming a locally homogenous EP distribution (“local homogeneity assumption”, LHA), EP value on each voxel can simply be calculated locally with obtained maps of the transverse *B*_1_ field components $B_{1}^{+}$ or $B_{1}^{-*}$ without the necessity of any assumptions about *B_z_*. Using a birdcage transceive RF coil and TPA, a local EPT solution is readily available. This approach has been adopted in some early EPT works [50]–[52], [67], [68].

It has been suggested that in the frequency range corresponding to MR field strength below 3 T, where the wave behavior of *B*_1_ is less significant, the absolute *B*_1_ phase depends on the conductivity distribution more than the permittivity while permittivity is more closely related to the magnitude of *B*_1_ fields [51], [67], [68]. Based on this observation, a phase-only conductivity reconstruction and a magnitude-only permittivity reconstruction method have been proposed as

(12) $$\sigma \approx \frac{\nabla^{2}\varphi^{\pm }}{\mu \omega }=\frac{\nabla^{2}\varphi }{2\mu \omega }$$

and

(13) $$\varepsilon \approx -\frac{\nabla^{2}|B_{1}^{\pm }|}{\mu \omega^{2}|B_{1}^{\pm }|}$$

where *φ*^+^ and *φ*^−^ are the absolute phase of $B_{1}^{+}$ and $B_{1}^{-*}$, respectively and *φ*:= *φ*^+^ *+ φ*^−^ is the transceive phase. The phase-only conductivity reconstruction is interesting because acquisition of phase data does not require additional *B*_1_-mapping procedures. Spin-echo [52], zero-echo-time [69] or steady-state-free-precession (SSFP) [70] sequences are less sensitive to background *B*_0_ distortion or chemical shift. The transceive phase can be estimated from such data without acquiring an additional *B*_0_ map, leading to reduced scanning time [70], [71].

**Fig. 4:**
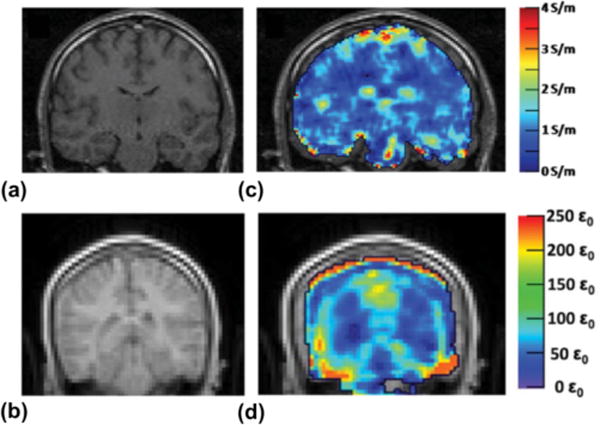
Results of reconstructed EPs of healthy human brain tissue *in vivo*. (a) and (b) Magnitude of MRI data for reconstruction of EPs in (c) conductivity and (d) permittivity, respectively. EPs were reconstructed using equation (10), magnitude *B*_1_^+^ and estimated phase based on TPA. Reproduced with permission from Voigt, Katscher and Doessel. Magn Reson Med 2011; 66:456–466.

Figure 4 exhibits results of EPs using the LHA with magnitude $\left|B_{1}^{+}\right|$ and TPA-estimated phase *φ*^+^ (Figs. 4c and d) [67]. This first *in vivo* study of EPT demonstrated its application for brain imaging based on LHA and TPA. The reconstructed conductivity shows reasonable value and colocalization with the gray/white matter and cerebrospinal fluid (CSF), while the permittivity result has a more severe problem, such as deviated anatomy compared with the reference MRI (Fig. 4b) and unrealistic values (> 200 *ε*_0_) particularly along the rim of the brain. This could be due to the large kernel size of the calculus operations smearing over different compartments [67].

The effectiveness of the introduced local solution so far can be challenged by several factors:

First of all, due to the violation against LHA, errors can happen near the interface between regions of different EP value [72], [73]. Tissues are usually characterized by complicated branching/frayed/fringed/fringy structures and thus spatially varying EP distribution. One way of improving the reconstruction near boundaries is to constrain the Laplacian operator within a region of ideally uniform EP value based on some reference image [67], [74], [75]. In practice, interpretation of the results using this strategy calls for caution because the reference image can potentially lead to bias in the EP reconstruction. Another approach is to use inhomogeneous equations such as (8) and (9) to construct the reconstruction algorithm. Using (8) and (9), the spatial gradient of ln *ε_c_* can be calculated to improve the reconstruction near boundaries [53], [63], [76]–[78]. This approach will be discussed later.

Secondly, the assumption of TPA becomes increasingly invalid at high field strength or with a non-quadrature RF coil. Increased field strength holds promises for improved intrinsic SNR and introduces more significant wave effect beneficial for EPT reconstruction. Moreover, high field MRI applications call for more accurate SAR estimation based on individual EP model and the current status of the RF-transmitting device [62], [79], [80]. At high (3 T) and ultrahigh (7 T) field, non-quadrature RF coils such as multi-channel parallel transmit RF coils or phased-array receive coils are beneficial for improved excitation coverage, SNR and accelerated acquisition [54], [55], [81]–[85].

**Fig. 5:**
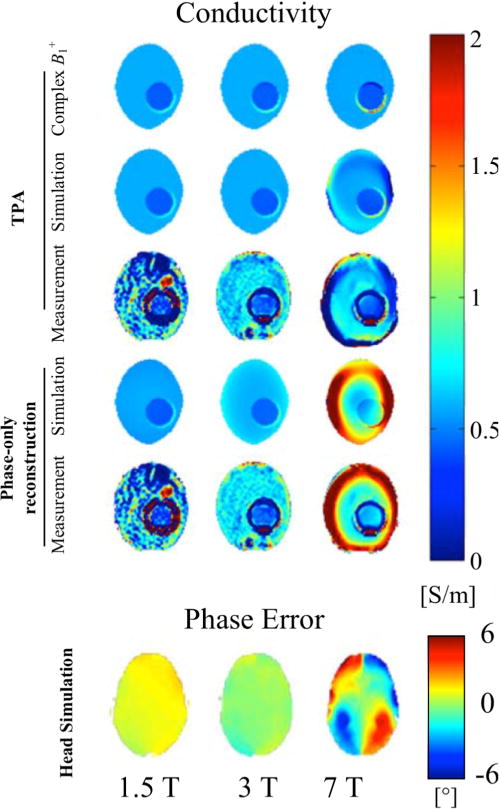
Top: TPA-based and phase-only conductivity reconstruction using measured and simulated *B*_1_^+^ data of a phantom (model) at different field strengths, respectively. Bottom: Error of TPA phase estimation based on simulated *B*_1_^+^ with a realistic head model at different field strengths. Reproduced with permission from van Lier et al. Magn Reson Med 2013; 71:354–363.

Van Lier et al. in 2013 has investigated the validity of the TPA assumption and phase-only conductivity reconstruction using quadrature birdcages RF coils at 1.5, 3 and 7 T, respectively. As shown in Fig. 5, the TPA assumption remains reasonably accurate at 1.5 and 3 T while introduces apparent phase error towards the peripheral of the head model and conductivity error in the phantom experiment and simulation both at 7 T. The conductivity estimation is even more severely disrupted if phase-only reconstruction is carried out.

Thirdly, local solutions are sensitive to noise in the measured *B*_1_ data and modeling errors. The effect of these error sources can be amplified through the Laplacian operator ∇^2^ in equations (8) and (9). Laplacian operation is effectively a high-pass spatial filter, and it amplifies spatial frequency harmonics to the second order of their frequency. To reduce the noise effect, a low-pass spatial filter can be applied on the *B*_1_ data before any reconstruction calculation. The resultant SNR of the reconstructed EPs is proportional to the square of the linear dimension of the effective kernel, which combines the Laplacian and the low-pass filter, and the square root of the number of voxels in the kernel [86]. However, using a low-pass filter will compromise the effective resolution. As will be discussed later in Section III.B, a global solution is helpful to reduce noise sensitivity of the local EPT algorithms without relying on a strong low-pass filter.

#### A.2. Local solutions Using Multi-channel B_1_ maps

The need for quantitatively calculating absolute *B*_1_ phase without relying on TPA or calculating EPs and their gradient using the inhomogeneous equations (8) and/or (9) motivates using multiple channels of RF elements to collect *B*_1_ data to solve for the additional unknowns. Fig. 3b is demonstrated as an example in which the magnitude of $\left|B_{1}^{+}\right|$ or $\left|B_{1}^{-*}\right|$ and the corresponding relative phase between different channels are acquired for calculating the unknown absolute phase of $B_{1}^{+}$or $B_{1}^{-*}$ of a reference channel [63], [87]–[89]. In addition, the transceive phase of a pair of transmit and receive channels can be obtained and used to solve the common unknowns, including EPs and absolute *B*_1_ phase [76], [90]–[92]. Note that $\left|B_{1}^{-*}\right|$measurement is usually weighted by the unknown proton density *ρ* in the form of $\left|\rho B_{1}^{-*}\right|$. Later, methods eliminating the effect of *ρ* will be discussed.

In [87], an equation about $B_{1}^{+}$ and $B_{1}^{-*}$ was derived based on the Gauss’ Law for magnetism and used to calculate absolute phase using measured relative phase and magnitude data. This approach requires that $B_{1}^{+}$ and $B_{1}^{-*}$ come from the same RF channel, and therefore, it is critical to accurately quantify any additional phase delay on the transmit or receive line and rescale the magnitude of $\left|B_{1}^{+}\right|$ and $\left|B_{1}^{-*}\right|$ on each channel based on a calibration. The works in [88], [90], [91] were based on the Helmholtz equations (10) and/or (11) using the relative and/or transceive phase data. In [89], a reconstruction algorithm was developed based on (11) and the relative sensitivity data of receive *B*_1_ channels. Relative receive sensitivity is inherently insensitive to non-*B*_1_^−*^-related components in the signal equation (4) such as T1, proton density and *B*_1_^+^. Additionally, it can be acquired efficiently with a single RF excitation. However, the concept is so far only implementable based on the Helmholtz equation, causing boundary errors, and the result can be sensitive to noise effect because the method relies on a third-order derivative to calculate EPs.

**Fig. 6:**
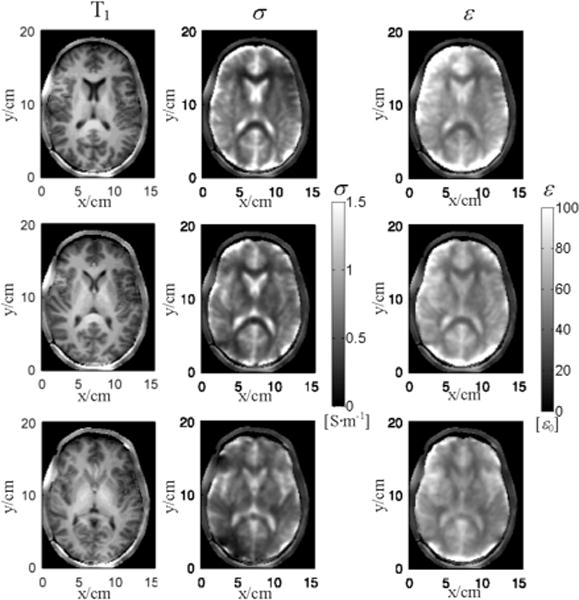
Reconstructed *in vivo* EPs using the gradient-based EPT algorithm in three slices of a healthy human subject’s brain. Left: reference T1-weighted images; middle: reconstructed conductivity images; right: reconstructed permittivity images. Reproduced with permission from Liu et al., 2015; 74: 634–646.

Based on the inhomogeneous equations (8) and (9), using multi-channel $\left|B_{1}^{+}\right|$, $\left|\rho B_{1}^{-*}\right|$ and relative phase of $B_{1}^{+}$ and $B_{1}^{-*}$, a gradient-based EPT method was proposed to first calculate a local solution of EP gradient and then obtain the final EP maps by integrating the gradient [63]. A typical result of brain EPT imaging is shown in Fig. 6. Because integration reduces high frequency noise, the result features enhanced robustness against noise contamination. The details of brain structure as revealed in the T1-weighted anatomical image can also be visualized in the images of conductivity and permittivity without disruptive error near boundaries between different tissues. The nominal in-plane resolution was 1.5 mm. With applied Gaussian filter, the effective resolution was around 5 mm. This work demonstrated the capability of EPT to achieve high resolution images of human brain *in vivo*. When $B_{1}^{-*}$ is considered, the unknown *ρ* needs to be first removed. In this and earlier studies [87], [90], *ρ* was extracted based on the symmetric pattern of $B_{1}^{+}$ and $B_{1}^{-*}$. It was shown later that, with measured transceive phase, *ρ* can be reconstructed quantitatively based on the transmit and receive *B*_1_ data [76]. In another study [92], it was suggested in theory that with sufficient *B*_1_ data, including magnitude, relative and transceive phase, and sufficient number of independent RF elements, a full model can be established to reconstruct absolute *B*_1_ phase, EPs and *ρ*, even without assuming *B*_z_=0. However, this model remains to be demonstrated to be feasible in practice due to its high numerical complexity and adverse noise figure.

### B. Global Solutions

Although the local solutions are straightforward to conceive and implement, they ignore the relations of EPs on neighboring voxels. As mentioned earlier, noise in the *B*_1_ data is amplified through the Laplacian operator ∇^2^. Solving the EPT reconstruction problem essentially derives a solution of partial differential equations (PDE) about EPs and *B*_1_ field. As shown in Fig. 2, in calculating a global solution, the *B*_1_ data from the entire ROI is provided to the solver, and EPs in all the voxels in the ROI are obtained together instead of one voxel after another. The relationship of EPs between neighboring voxels is automatically considered in the model. The aforementioned gradient-based EPT can be considered as a hybrid method, with a local solution of gradient and global solution of final EPs based on the calculated gradient.

A global EP solution can be obtained based on backward calculation from the measured *B*_1_ data to reconstructed EP values. This approach was first realized in the so-called crMREPT method [77]. Replacing $\frac{1}{\varepsilon_{c}}$ with *λ*, equation (8) is transformed to a linear PDE [77], [78]

(14) $$-\omega^{2}\mu_{0}B_{1}^{+}=\left(\frac{\partial B_{1}^{+}}{\partial x}-i\frac{\partial B_{1}^{+}}{\partial y}\right)\left(\frac{\partial \lambda }{\partial x}+i\frac{\partial \lambda }{\partial y}\right)+\frac{\partial B_{1}^{+}}{\partial z}\cdot \frac{\partial \lambda }{\partial z}+\nabla^{2}B_{1}^{+}\lambda$$.

In the crMREPT study, *λ* was calculated globally based on a measured transmit *B*_1_ magnitude and TPA-estimated absolute transmit *B*_1_ phase using the finite-element method. From the same group, a phase-only gradient-based global solution of conductivity has been proposed derived from (14) [78].

**Fig. 7:**
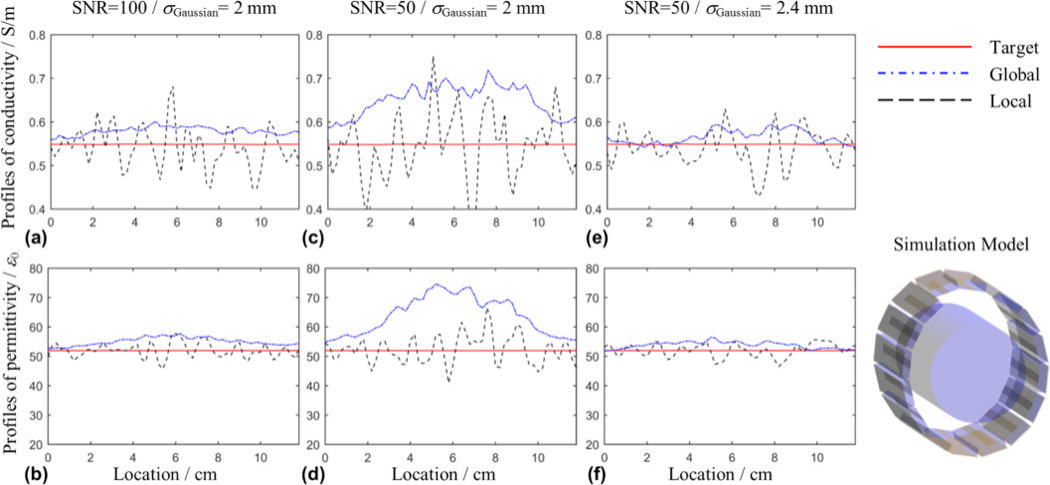
A comparison of noise effect on EPT reconstruction between a global and local solution based on a finite-difference time-domain simulation using a 16-channel microstrip RF array coil [92] loaded with a cylindrical homogeneous phantom model (*σ*: 0.55 S/m and *ε*: 52 *ε*_0_) at 7 T.

As shown in Fig. 7, we used simulated $B_{1}^{+}$ data based on finite-difference time-domain (FDTD) method to demonstrate the effect of noise on an example of global solution in comparison with a local solution. Complex $B_{1}^{+}$ from sixteen channels of the RF array coil was generated with a homogeneous cylindrical dielectric model with isotropic 2-mm resolution. Gaussian noise was added to the real and imaginary parts of $B_{1}^{+}$ with a spatially constant standard deviation (SD). SD was 1/SNR times the average $\left|B_{1}^{+}\right|$ in the dielectric model. The noise-contaminated data was first filtered with a 3D low-pass Gaussian filter with a standard deviation of *σ*_Gaussian_. The global solution was constructed based on (14) and the finite-difference method [93] within a xy-plane, with eliminated $\frac{\partial B_{1}^{+}}{\partial z}\cdot \frac{\partial \lambda }{\partial z}$ and using the noise-contaminated sixteen $B_{1}^{+}$ data sets. The local solution was calculated based on (10) using the same $B_{1}^{+}$ data. Note that since the model is homogeneous, using the Helmholtz equation (10) is justified. In Figs. 7a and 7b (SNR=100), it can be observed that the results using the global solution show less fluctuating profiles compared to the local solution results, with the latter swinging about the target value of the simulation input and the former globally biased. At a lower SNR of 50, using the same *σ*_Gaussian_ as that in Figs. 7a and 7b, both the fluctuation of the local solution and global bias of the global solution become more significant as shown in Figs. 7c and 7d, especially near the center of the model. Increasing *σ*_Gaussian_ of the filter removes a larger portion of the random noise and improves the performance of the both methods as shown in Figs. 7e and 7f. In general, it can be observed that the global solution can significantly reduce the fluctuating effect of the noise because the relationship of EPs between neighbor voxels are used as additional information to reduce the Laplacian-and-derivative amplified noise fluctuation. However, the global solution has the side effect of a systematical global bias around the center of the model. In this example, $B_{1}^{+}$ data from all the channels tends to have a very low magnitude of $\left|\frac{\partial B_{1}^{+}}{\partial x}-i\frac{\partial B_{1}^{+}}{\partial y}\right|$ around the center, reducing the efficacy of the global solution around the central region. A similar problem has been observed in the crMREPT study, in which artifacts arise around the region with low $\left|\frac{\partial B_{1}^{+}}{\partial x}-i\frac{\partial B_{1}^{+}}{\partial y}\right|$.

A global solution can also be derived based on a forward model in which the complex *B*_1_ distribution is calculated for specific EPs with a RF coil model or certain boundary conditions about *B*_1_; the EP solution is updated iteratively to minimize the difference between calculated *B*_1_ distribution in the forward model and the measured *B*_1_ data. In the contrast source inversion (CSI) EPT study [94], the total field was modeled as the sum of the incident field in an empty coil and the secondary scattered field when the coil is loaded with the object. This method does not need any assumption about *B_z_* distribution. However, accurate forward calculation of the incident magnetic and electric field with an accurate model of the RF coil and consideration of scattering effect inside the MR bore is quite challenging. Another similar approach that was also formulated based on the scattering theory can be found in [95]. In the study of [96], the dubbed global maxwell tomography method iteratively reconstructs EPs based on a full model of the RF coil and the subject until the simulated $\left|B_{1}^{+}\right|$ is sufficiently close to the measured map. It faces the similar challenge of the demanding modelling accuracy as the CSI-EPT method. Instead of a full model of the RF coil, in [97], forward calculation of $B_{1}^{+}$ was based on (8) and simulated $B_{1}^{+}$ (or measured data in experiment) on boundary of the ROI assuming *B_z_*=0.

Global solution can be constructed by introducing regularization methods to improve the image quality which can be hampered by the intrinsic limitation of EPT, such as noise amplification, missing *B*_1_ components, violation of LHA, etc. In the CSI-EPT method, improved robustness against noise was shown using a multiplicative total variance regularizer. In a recent work [98], different regularization strategies were applied to regions of smooth EP distribution and regions of rapid EP changes, respectively, with the former aimed at reducing amplification of random noise and modeling error and the latter preserving the edge of tissue EPs. In contrast to the global methods which rely on the inhomogeneous equations such as (8) or (14), formulations utilizing the simplified, LHA-based equations such as (12) and regularization schemes have been proposed and shown with improved performance near boundaries and reduced sensitivity to noise [99], [100].

### C. Spatial and Temporal Resolutions

In an ideal situation when the *B*_1_ data is free of thermal noise, spatial resolution of the reconstructed EP image using EPT depends on the resolution of the measured *B*_1_ map, preprocessing method and reconstruction algorithm. In a previous study [76], profiles of the reconstructed EPs using the full equations (8) and (9) and simulated noise-free *B*_1_ data suggest the same resolution of the reconstruction as the input *B*_1_ data. In practice, when a low-pass filter is used to reduce the noise effect, the effective resolution will depend on the filter. For example, a Gaussian filter with a standard deviation of *σ*_g_ voxels will result in an effective resolution of 2.67 *σ*_g_ based on the full width of half maximum (FWHM) of the Fourier spectrum of the filter [63]. The level of smoothing is driven by the desired contrast-to-noise ratio (CNR) in the reconstructed EPs. Analysis of the noise level in the reconstructed EPs suggests that it is related to the field strength, FWHM (in unit of length rather than voxels) of the low-pass filter and number of measured voxels in the kernel of low-pass filter and Laplacian [86]. By considering the integrative effect of these factors, so far a resolution between 3 and 5 mm has been achieved in the reconstructed EP image [63], [101], [102].

Temporal resolution of EPT methods varies as the *B*_1_ data required for different reconstruction algorithms differs. The phase-based conductivity reconstruction based on (12) or proposed in [78] only utilizes the transceive phase which can be acquired with a fast, *B*_0_-insensitive SSFP sequence. A time frame of 4 s of conductivity imaging based on (12) has been reported [70]. The approach [89] based on the relative coil sensitivity also has the advantage of a high temporal resolution even though the original paper was published using the data acquired with a slower GRE sequence.

## IV. Biomedical Applications of EPT

### A. Cancer diagnosis, staging and grading

Tumorigenesis is accompanied by significant local changes at molecular, cellular and tissue levels. For example, sodium concentration and water content are elevated due to aggressive proliferation; nucleus takes a much larger volume of the cell body and cytoplasm becomes much denser; cellular density substantially increases with extracellular matrix impaired to facilitate cancer invasion. These abnormalities also develop as tumor grows into various stages. EPs, as fundamental physical properties of biological tissues, correlate with the tumor features and represent responsible and responsive indicators for cancer diagnosis, staging, and grading.

It is noteworthy that a huge variation of tissue EPs exists across the body, such that contrast between normal and cancerous tissue is dependent not only on tumor itself but also the properties of its surrounding tissues [9]. This heterogeneity of contrast plays a fundamental role when determining the sensitivity and specificity of EPT to pinpoint cancer in different organs.

#### A.1 Breast Cancer

Utilizing different implementations of the polynomial fitting method of turbo spin echo or fast spin echo phase maps, several pilot studies have demonstrated significant conductivity elevation in malignant lesions compared to normal breast tissues and benign cysts [74], [103], [104]. A retrospective study involving 65 female patients with invasive breast cancer further revealed the correlation between reconstructed conductivity and prognostic information such as tumor type and status [105]. The clinical applicability of EPT is still pending establishment, potentially through large-scale prospective studies including various subtypes, sizes, and stages of breast lesions.

#### A.2 Other Cancer Types

**Fig. 8:**
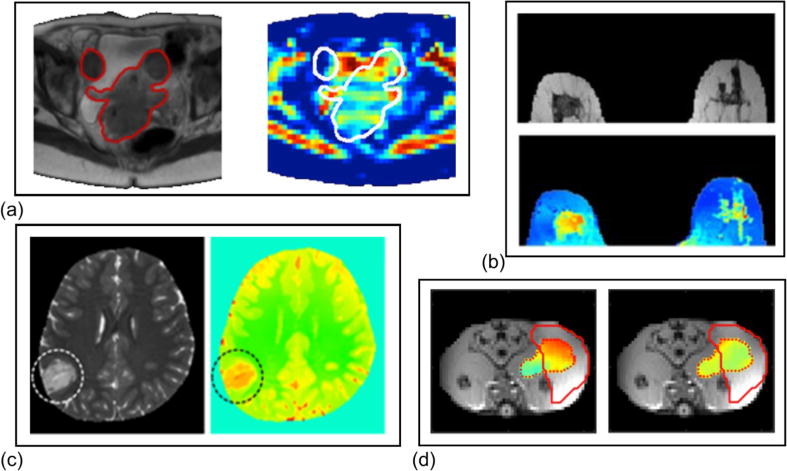
(a) Cervix tumor delineated on anatomical image and projected onto conductivity map. Reproduced with permission from Balidemaj et al., Magn Reson Med 2015; 73:1505–1513. (b) Anatomical and conductivity images of the breast of a tubular carcinoma patient. Reproduced with permission from Shin et al., J Magn Reson Imaging 2015; 42:371–378. (c) Dysembryoplastic neuroepithelial tumor showing hyper-intensity on SSFP and its conductivity distribution [110]. (d) Conductivity and permittivity overlaid on proton density-weighted image of a rat tumor xenograft model. Reproduced with permission from Liu et al., Magn Reson Med 2017.

EPT can also be applied to examine other organs for cancer characterization. As shown in Fig. 8a, in the pelvis, feasibility of conductivity measurement in cervix tumor has been demonstrated in 20 patients [106], [107]. In the brain, conductivity of glioma has been reported to deviate from surrounding healthy tissue [108], [109]. This deviation varies with tumor type and positively correlates with tumor grade, which is important reference information for therapy design, and was reported to be consistent with probe measurements of excised specimen [110], [111].

#### A.2 Animal Cancer Models

Recently, tumor-bearing rodent models have been used in several EPT studies. Animal models have the unique advantages of better availability and flexibility, opening the possibility for more thorough, well-controlled investigations to correlate EPs with cancer features.

An animal tumor study in the field utilized combined *B*_1_ magnitude and transceive phase from a quadrature coil at 3T [112]. Both conductivity and permittivity of implanted adenocarcinoma on three rats are reported to be consistent with probe measurements. In another study, Liu et al. successfully demonstrated the conductivity difference between implanted tumor and the surrounding muscle with high sensitivity as shown in Fig. 8d [101]. This study utilized a dedicated 8-channel microstrip transceiver array coil developed for 7T MRI and reconstructed with the gradient-based EPT algorithm [63]. Future studies are expected to take on animal models bearing more resemblance to human cancer and to monitor tumor development along time course.

### B. Subject-specific tissue mapping

EPs of normal tissues have been investigated and tabulated using invasive dielectric probes [113], [114]. The Cole-Cole fitting has been performed based on this data, and the resultant curves are widely used in numerical modeling for SAR estimation and thermal dose computation in hyperthermia [5], [115]. However, organs are intrinsically heterogeneous in composition and structure; their EPs also vary with age [116], [117] and short-term biological effects such as blood supply [118]. Additionally, based on EPT-measured *in vivo* conductivity of muscle in 20 cervical cancer patients, Balidemaj et al. reported a 14% systematic deviation from the Cole-Cole-fitted curve and significant inter-subject variability [107]. These data suggest the necessity of subject-specific EP mapping for precision diagnosis and treatment at personalized level.

### C. Other Applications

#### C.1. In the Brain

A case study of an ischemic stroke patient at 7 T reported that conductivity in the infarction was elevated by a factor of more than two [119]. Another study involves two cases of hemorrhagic and ischemic strokes patients in subacute stage [120], using the recent phase-only EPT reconstruction algorithm that alleviates tissue boundary artifacts [78]. They reported increased conductivity in ischemic lesions and edema, but not hematoma itself, hypothetically due to clot formation.

Taking *a prior* knowledge from quantitative magnetic susceptibility maps for EPT reconstruction, another study investigated conductivity of deep brain nuclei [121]. The reconstructed conductivity and susceptibility are reported to be uncorrelated, suggesting that EPs can provide an additional dimension of information about these important brain structures.

#### C.2. In the Body

For body imaging, the major challenges fall into the experimental part to obtain reliable *B*_1_ magnitude and phase distributions that are minimally affected by confounding factors, such as *B*_0_ inhomogeneity and motion artifacts. Most previous studies assumed homogeneity of *B*_1_ magnitude in a relatively small ROI and applied phase only conductivity imaging to various organs in the body. SSFP sequence is favorable in these applications due to its insensitivity to *B*_0_ effects and fast speed.

Except for the cervix tumor imaging discussed above, there are three more instances where EPT was utilized to retrieve the conductivity of different body organs, showing the broad spectrum of the potential applications: (1) The livers of 10 healthy subjects were examined using SSFP during inspiration and expiration, leading to conductivity measurements that are consistent with published values [71]. (2) Two isolated perfused pig heart models, one of which with induced severe ischemia, were scanned using gated SSFP, reporting conductivity values of normal tissue close to previous literature and roughly 60% decrease in infarcted area [122]. (3) Five healthy volunteers were recruited for a lung scan, and the expected conductivity difference between inspiration hold and expiration hold was observed [123]. An ultrashort-TE (UTE) sequence was used in this study so as to address the fast signal decay in the lung due to short T2 and severe *B*_0_ inhomogeneity.

#### C.3. Inferring Electrolyte Concentration

At the Larmor frequency range (>100 MHz), it is expected that the charge-barrier effect of cell membrane on conductivity is weak and conductivity can be directly influenced by ion concentration. Studies in [124] and [125] examined and suggested a linear relationship between sodium concentration measured using sodium MRI and EPT-based conductivity value *in vivo* among different brain tissues. In a more recent study [126], it was suggested that the portion of free, non-protein-bounded sodium should be accounted for to estimate the conductivity value. Based on these preliminary results, conductivity image measured based on EPT can potentially be used to extract quantitative sodium information while traditional sodium MRI has been hindered by its limited SNR, need of special RF coil and sequence.

Studies also attempted to establish a relationship between water content and EPs in the Larmor frequency range. This effort was done in a study based on protein solution [127] and more recently in *in vivo* MRI measurement [128]. Because increased water content can lead to increased total ion density assuming a constant ion concentration, the unique role of water in defining the conductivity value is not clear. Caution is needed when these models are used to predict conductivity of abnormal tissues which may have an altered ion concentration, e.g. increased sodium in tumor tissue[129].

## V. Application of EPT in Prediction of Subject-specific SAR

Local SAR relates to RF-induced local heating of tissue during an MRI scan. Local SAR is required to be determined for any RF coil and RF pulse to be applied to scan the subject. As mentioned earlier, current practice of estimating SAR based on generic human models does not account for the operating status of the RF transmit device or the specific subject’s EP distribution. EPT can play an important role in subject-specific SAR estimation because it automatically provides the subject-specific EPs and RF distribution under its framework.

Local SAR is given by

(15) $$\text{SAR}=\frac{\sigma {\left|E\right|}^{2}}{2\rho_{m}}=\frac{\sigma }{2\rho_{m}}{\left|\frac{\nabla \times B}{\omega \mu_{0}\varepsilon_{c}}\right|}^{2}$$

where *ρ_m_* is the tissue mass density. Expressing **E** via the magnetic field **B** using Ampère’s Law as done in (15), one can find that determination of local SAR requires not only conductivity, but also permittivity. The determination of conductivity and permittivity is discussed in the previous sections, and are assumed as known quantities in this section. Tissue density is not directly accessible with MR imaging, but is sufficiently constant throughout the body and can be approximated by the density of water around 1g/cm^3^ (e.g., deviation from muscle and fat tissue is around 5%).

The remaining problem of solving (15) is thus the determination of **B**. In contrast to the determination of EPs, which can be derived exactly from a single spatial component of **B** (assuming constant EPs in (3)), local SAR cannot be derived exactly from a single spatial component of **B**, even in areas with constant EPs. The MR accessibility of the spatial components *B*_1_^+^, *B*_1_^−*^, which are equivalent to *B_x_*, *B_y_*, as shown in (5), and *B_z_* are summarized in Table I, separately regarding their magnitude and phase. From these six quantities, only the magnitude of *B*_1_^+^ is directly measurable via *B*_1_ mapping as discussed above. Three further quantities (magnitude of *B*_1_^−*^ as well as phase of *B*_1_^+^ and *B*_1_^−*^) are influencing the MR signal expressed by (4) but cannot be measured directly. Magnitude and phase of *B*_z_ do not influence the MR signal at all. Different methods about how to estimate the missing quantities are a focus of several studies on EPT-based SAR determination, as discussed in the following.

An additional issue is the scaling of **B**, which cancels out for the calculation of EPs as shown in (8) and (9), and thus, is not required to obtain EPs quantitatively. Such cancellation is not given in (15), and thus, the quantitative determination of local SAR requires the knowledge of the scaling of **B**. Fortunately, this scaling can be obtained in a straightforward manner by comparing measured flip angle, applied RF pulse shape and duration.

Studies on EPT-based local SAR determination are frequently performed with quadrature birdcage coils for head or whole body. Particularly for the central axial plane of such a coil, a couple of assumptions can be applied, which simplify (15) significantly. First, *E_z_* is the dominant component in this plane, and *E_x_* and *E_y_* can be neglected. Calculation of *E_z_* does not depend on the unmeasurable *B_z_*, but only on *B*_1_^+^ and *B*_1_^−*^. Moreover, assuming $\frac{\partial B_{z}}{\partial z}=0$, *E_z_* =0 can be expressed as a function of *B*_1_^+^ only [62], [130]

(16) $$|E_{z}|\approx \frac{2}{\left|\omega \mu_{0}\varepsilon_{c}\right|}\left|\frac{\partial B_{1}^{+}}{\partial x}-i\frac{\partial B_{1}^{+}}{\partial y}\right|$$.

Thus, *B*_1_^+^ enables a satisfying estimation of local SAR determination in the central transverse plane of a quadrature volume coil. The determination of *B*_1_^+^ can be performed as described in the previous sections for the determination of EPs.

For determination of local SAR of a quadrature volume coil, *B*_1_^−*^ can be treated as zero since $|B_{1}^{+}|>>\left|B_{1}^{-*}\right|$ when the RF coil is in the transmission mode. This is not the case for imaging, where the coil is switched from RF transmission to RF reception mode yielding $|B_{1}^{+}|\approx \left|B_{1,\text{switched}}^{-*}\right|$, which enables the absolute *B*_1_ phase estimation based on TPA as introduced in Section III.A.

**Fig. 9:**
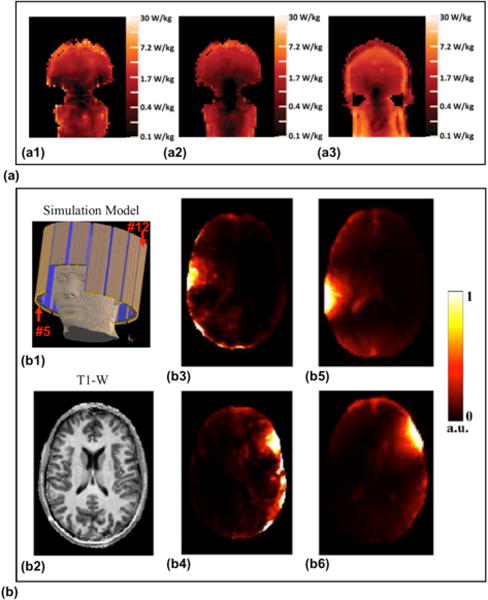
EPT-based local SAR estimation under *in vivo* setups. (a) Local SAR distribution with a quadrature birdcage RF coil at 1.5 T: (a1) local SAR estimation based on measured complex *B*_1_^+^ data; (a2) local SAR estimation using the phase-only method; (a3) simulated SAR distribution. (b) Local SAR distribution of individual channels with a 16-channel microstrip transceive RF array coil at 7 T: (b1) the simulation model showing the RF coil; (b2) a T1-weighted image of the imaged slice; (b3) and (b4) local SAR estimation of channel #5 and #12, respectively; (b5) and (b6) simulated local SAR of the two channels. (a) was reproduced with permission from Voigt et al., Magn Reson Med 2012; 68:1117–1126, and (b) was reproduced permission from Zhang et al., IEEE Trans Med Imaging 2013; 32:1058–1067

EPT-based local SAR determination has first been studied by phantom experiments using quadrature excitation at 1.5 T, using the assumptions outlined above [52]. The first corresponding *in vivo* results were obtained with the same experimental setup [80], i.e., using quadrature excitation at 1.5T. Shown in Fig. 9a is the local SAR estimation in a middle slice in this study, based on the complex *B*_1_^+^ data in Fig. 9a(1) or phase-only data in Fig. 9a(2), in comparison with simulated SAR based on the model of the same setup. Shortly after, the concept was extended to non-quadrature single elements of a transmit coil array at 3T [90], [130] by quantitatively estimating absolute transmit *B*_1_ phase using the MR-measurable multi-channel *B*_1_ data instead of relying on the TPA assumption. In contrast to [90], [130] based on the Helmholtz equation, [62] was based on the full equation (8), deriving absolute transmit and receive phases of each coil element by Gauss’s Law and the extracted proton density at 7 T. The results in this study are illustrated in Fig. 9b, showing consistency between the estimated single-channel local SAR in a healthy subject in reference to the FDTD simulation data. It has been repeatedly shown that after determination of electric fields of the single transmit array elements, the prediction of local SAR for arbitrary *B*_1_-shim settings is possible [130], [131].

Neglecting derivatives of *B_z_* (which is done in all studies mentioned above) is reasonably valid for volume coils as outlined in the previous sections, but less valid for the upcoming type of transmit arrays consisting of local elements (“mattresses”), particularly near transversely oriented parts of conductors. On the other hand, patient-individual SAR determination is of increased interest for these local transmit arrays due to their individual placement on the patient and high-degree-of-freedom RF pulse design. A fast and simple way to estimate *B_z_* for local transmit arrays is possible by integrating *B*_1_^+^ and *B*_1_^-*^ via Gauss’s Law [132] as

(17) $$B_{z}(x,y,z)=-\frac{1}{2}\int_{z_{0}}^{z}\left(\begin{array}{l} \frac{\partial B_{1}^{+}(x,y,z^{\prime })}{\partial x}-i\frac{\partial B_{1}^{+}(x,y,z^{\prime })}{\partial y}\\ +\frac{\partial B_{1}^{-*}(x,y,z^{\prime })}{\partial x}+i\frac{\partial B_{1}^{-*}(x,y,z^{\prime })}{\partial y} \end{array}\right)dz^{\prime }.$$

This approach requires that the field of view (FOV) of the measured *B*_1_^+^ and *B*_1_^-*^ can be extended in feet-head direction to *z*_0_ with *B*_1_^+^(*z*_0_) ≈ *B*_1_^-*^(*z*_0_) ≈ 0, which is a realistic task for the local surface coils regarded. In contrast, local SAR occurring outside the maximal FOV is a potential drawback investigating transmit body coils.

Once the spatial distribution of the electrical tissue properties is determined, a forward model can be applied to estimate (all spatial components of) the required fields. For EPT-based SAR determination, this has been realized by [133] in the framework of the aforementioned CSI-EPT. However, as mentioned above, a forward model is of considerable effort since it requires not only a model of the subject, but also of the RF coil involved. At least, in contrast to the subject model, the coil model is known *a priori* from the coil’s manufacturing. A coil model enables furthermore the determination of the conservative part of the electric field, which is not possible by purely post-processing the measured magnetic field as in (15). Conservative electric fields (obeying Gauss’s law and curl-free) are generated predominantly by electronic components of the RF coil, but might be negligible within the body of subject due to their rapid decrease with the distance from the components.

The ultimate goal for RF safety management is the prediction of RF-induced, local tissue temperature increase. In the framework of EPT-based SAR determination, this challenge has been investigated in [131], [134]. It has been shown that prediction of temperature increase is possible, particularly taking heat diffusion into account [134]. Both studies are based on phantoms, justifying the disregard of physiologic temperature regulation, which will become mandatory for corresponding *in vivo* studies. As shown in Fig. 10 from [131], the temperature increase of a specific *B*_1_-shimming mode is predicted by the estimated electric field of individual transmit elements in the EPT framework and validated with the MR thermometry measurement.

## VI. Summary

Electrical properties of biological tissue are related to a broad spectrum of parameters and can be used as biomarkers for diagnosing and monitoring diseases such as tumor, stroke, liver cirrhosis, etc. EPs are also widely used in understanding the interaction of tissue with electromagnetic field for improving the efficacy and accuracy of electromagnetic stimulation or recording in biomedical applications. Much effort has been dedicated to non-invasive and *in vivo* measurement of EPs. Electrical properties tomography has been developed to reconstruct the distribution of EPs based on the MR-measured distribution of *B*_1_ field, which is inherently used in MRI scanners. The high spatial resolution and simple operation on the subject make EPT an interesting and potentially important method for clinical applications related to EPs.

With increasing interest in the field, EPT has gained rapid growth in recent years. Following original demonstration of its feasibility, most effort has been devoted to overcoming the challenges of EPT, including boundary error in the Helmholtz equation-based methods, sensitivity to measurement noise and unmeasurable *B*_1_ component such as its absolute phase and *B_z_*. A fundamental challenge of EPT is that only partial information of the *B*_1_ field can be measured (Table I) so that assumptions are needed for calculation of EPs based on Maxwell’s equations. In Section III, we have reviewed major algorithm developments aiming to address these challenges. Among the development, multichannel *B*_1_-based algorithms were proposed for calculating both absolute *B*_1_ phase and EPs with reduced assumptions about the *B*_1_ field, inhomogeneous equations (8) and (9) were used to improve the performance near boundaries, and global solutions were shown promising for addressing both noise sensitivity and boundary issues.

EPT methods have been applied to detect the EP change in diseases such as brain/breast/pelvic tumors, animal tumor models, stroke, etc. Promising results have been reported by comparing with other imaging modalities or validated by probe measurement of EPs. Local solutions using TPA-based phase estimation or phase-only conductivity reconstruction have been the power horse in applications involving patients. This is partially owing to the accessibility of quadrature birdcage RF coils in clinical scanners, available phase data using existing protocols, short scan time and less complicated local Helmholtz-based reconstruction algorithms. Adaptive Laplacian kernel to a reference contrast image with local polynomial fitting was used to avoid boundary errors, at the cost of potential adverse effect from the selected reference image. Future applications should include large-scale studies to demonstrate the value of EPT for a specific subtype of disease in comparison with other existing imaging modalities. For example, EPT can potentially play an important and unique role in detection of tumor in the early stage or differentiation between benign and malignant tumor. At current stage, efforts from multiple research groups on a specific disease model are valuable to demonstrate the clinical impact of EPT methods. Future applications utilizing advanced EPT methods such as multi-channel-based reconstruction or global solutions are anticipated to achieve high resolution without the potential bias of a reference image.

Fast and subject-specific SAR estimation is an important application of EPT at high field strength. The dynamic SAR estimation can in principle be used to optimize RF pulse design and further MRI development with less conservative RF safety constraints. At higher field, it drives the development of multi-channel EPT methods as the assumptions originally adopted in the early development of EPT are not valid anymore and multi-channel RF transmission becomes common. It is critical to restore the missing field component of *B_z_* for certain RF coils with current running in perpendicular to the *B*_0_ direction. Temperature is the ultimate factor that determines RF safety. Therefore, more accurate heat transfer models need to be developed, considering the estimated SAR, heat transfer rate of different tissues and the convection effect of blood, etc.

## Figures and Tables

**Fig. 10:**
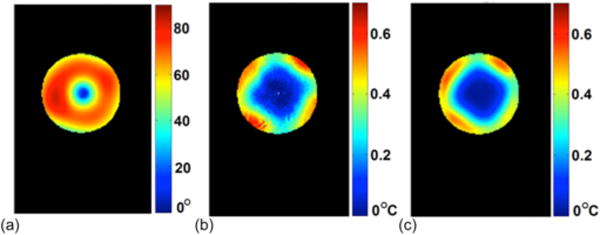
Prediction of temperature increase due to the RF-induced heating in a transmit array coil. (a) Induced flip angle under the tested *B*_1_-shimming mode. (b) Predicted temperature change due to RF heating with the tested *B*_1_-shimming mode in (a). (c) Measured temperature change using MR thermometry. Reproduced with permission from Zhang et al., Appl Phys Lett 2014; 105:244101.

**TABLE I:** Summary of MR accessibility of the different components of **B**

	Magnitude	Phase
*B*_1_^+^	“Direct” measurement	“Indirect” measurement
*B*_1_^−^	“Indirect” measurement	“Indirect” measurement
*B*_z_	No measurement possible	No measurement possible
